# Cardiac Lymphoma Diagnosed by Multi-Modality Imaging: A Case Report

**DOI:** 10.3389/fcvm.2022.771538

**Published:** 2022-04-08

**Authors:** Dayan Yang, Tangna Wu, Lini Gao, Lili Liu, Fujin Liu, Xiangxiang Jing

**Affiliations:** ^1^Department of Ultrasonography, Hainan General Hospital (Hainan Affiliated Hospital of Hainan Medical University), Haikou, China; ^2^Department of Pathology, Hainan General Hospital (Hainan Affiliated Hospital of Hainan Medical University), Haikou, China

**Keywords:** myocardial contrast echocardiography, cardiac lymphoma, diagnosed, ultrasound, cardiac tumor, multi-modality medical imaging

## Abstract

A 79-year-old female patient who presented with a cardiac mass detected by conventional echocardiography was ultimately diagnosed with a malignant tumor by myocardial contrast echocardiography. A positron emission tomography/computed tomography examination showed tumors in the right atrium consistent with the findings of the contrast-enhanced ultrasound. Finally, the patient was confirmed by pathology to have cardiac lymphoma. Because no lesions were found elsewhere in the body, primary cardiac lymphoma was diagnosed by combining multi-modal imaging examination and pathological examination. Although conventional echocardiography may identify a cardiac mass, it is difficult to identify whether they are malignant or not. Myocardial contrast echocardiography helps to identify the location, shape, and size of the mass, its relationship with the surrounding tissue, and evaluate its blood supply. Thus, this imaging modality is of great value for identifying the likely etiology of a cardiac mass. Multi-modal imaging is complementary to echocardiography for determining the location of cardiac masses, invasion of surround structures, extra cardiac spread, and determination of whether a mass is likely benign or malignant. Multi-modality imaging provides an important basis for clinical treatment and decision-making.

## Introduction

Primary cardiac lymphoma is a very rare extranodal lymphoma. Non-Hodgkin's lymphoma is the diagnosis for 80–90% of all lymphoma patients, with one-third of tumors diagnosed in lymphoid tissue outside the lymph nodes or lymphoid tissue organs of agglomeration. Lymphoma can occur in any part of the body with different clinical manifestations, although its physical signs are mostly primary organ enlargement or local tumor formation. Primary cardiac lymphoma accounts for 1% of primary cardiac tumors and 0.5% of extranodal lymphomas (1). The final diagnosis of cardiac lymphoma is determined by pathology, which causes some trauma to the patient and has certain limitations. In addition, cardiac imaging can also provide some valuable information, such as the mass's location, shape, and size.

Here, we present the case of a 79-year-old female patient who had a cardiac mass detected by conventional echocardiography (ECG). She was diagnosed with a malignant tumor by myocardial contrast ECG, to which pathology confirmed the tumor was a cardiac lymphoma.

## Case Description

A 79-year-old female patient had been complaining of chest pain for over 2 weeks. She described a tingling in the pre-cardiac area that was unrelated to breathing or activity, and could not be relieved by rest. She reported no radiating pain. Occasionally, the patient experienced night sweats, fatigue, acid reflux, and belching. Nausea, vomiting, and abdominal pain were also present. The vomiting of the gastric contents ultimately resolved itself. Before presentation, however, she had a continuous fever for 4–5 days with a peak body temperature of 38.5°C, which was occasionally accompanied by a dry cough. Self-reported use of oral antipyretics reduced her temperature to normal. The patient experiences lack of sleep, poor appetite, and a poor mental state. She had a history of cerebral infarction. No obvious change in body weight had recently occurred. The patient denied a family history of high blood pressure, diabetes, and coronary heart disease.

The patients physical examination upon admission revealed a temperature of 36.4°C, pulse rate of 103 bpm, breathing rate of 20 breaths/min, and blood pressure of 99/75 mmHg. Physical examination revealed that her superficial lymph nodes were not enlarged. There were no abnormal heaves. The apex beat was 0.5 cm inside the midline of the fifth intercostal space. No cardiac murmurs or rubs were heard and no other abnormalities were found during the rest of the examination. A blood routine examination revealed no obvious abnormality. Her white blood cell count was 5.01 × 10^9^/L, lymphocyte percentage was 19.4%, red blood cell count was 3.77 × 10^12^/L, hemoglobin concentration was 109 g/L, and platelet count was 71 × 10^9^/L. Multi-tumor marker protein chromatin immunoprecipitation (ChIP) detection (female, 12 items) showed no special abnormalities. Her ferritin concentration was 529.08 ng/ml, and the values of five key cardiac enzymes were as follows: aspartate aminotransferase, 149.6 U/L; lactate dehydrogenase, 809.0 U/L; α-hydroxybutyrate dehydrogenase, 602.0 U/L; phosphocreatine kinase isozyme, 27.4 U/L; and C-reactive protein, 37.26 mg/L. Her 72-h blood bacterial culture was negative. Her erythrocyte sedimentation rate was measured, and three results of the tuberculosis antibody ChIP, four results of coagulation, four results of blood transfusion, and liver and kidney functions were all normal.

**Figure 1:**
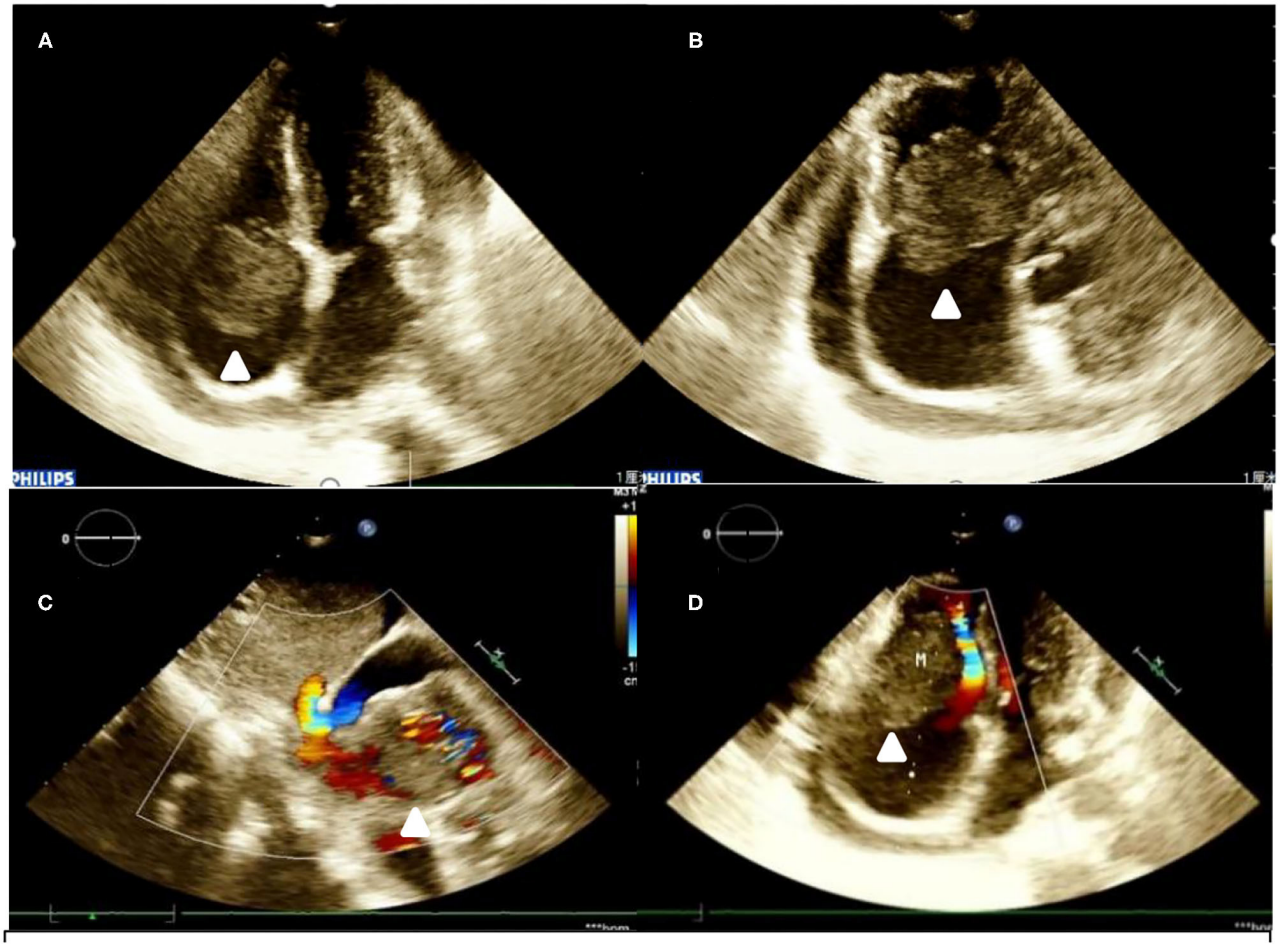
Transthoracic echocardiogram. **(A,B)** A hypoechoic mass is seen at the base of the right atrium lateral wall. It is highly mobile and oscillates through the tricuspid valve in diastole. **(C)** Color Doppler flow imaging in subcostal two-chamber view. **(D)** Color Doppler flow imaging in four-chamber view).

Conventional ECG revealed that the patient's right atrium was enlarged, and a hypoechoic mass (40 mm × 39 mm) was found to be connected to the bottom of the lateral wall. The mass appeared to oscillate with the cardiac cycle in the tricuspid valve orifice, causing right ventricular inflow tract stenosis in diastolic periods. The right and the left ventricles were both roughly normal in size. A large amount of pericardial effusion was detected (Figures 1A–D). Subsequently, an IE33 ultrasound machine (Philips, Amsterdam, Netherlands), of which the probe frequency was 2.5 MHz, was used to obtain myocardial contrast ECG images. An ultrasound contrast agent was purchased from SonoVue (Bracco, Italy) and was reconstituted by adding 5 ml of 0.9% sodium chloride solution, then injected intravenously.

**Figure 2:**
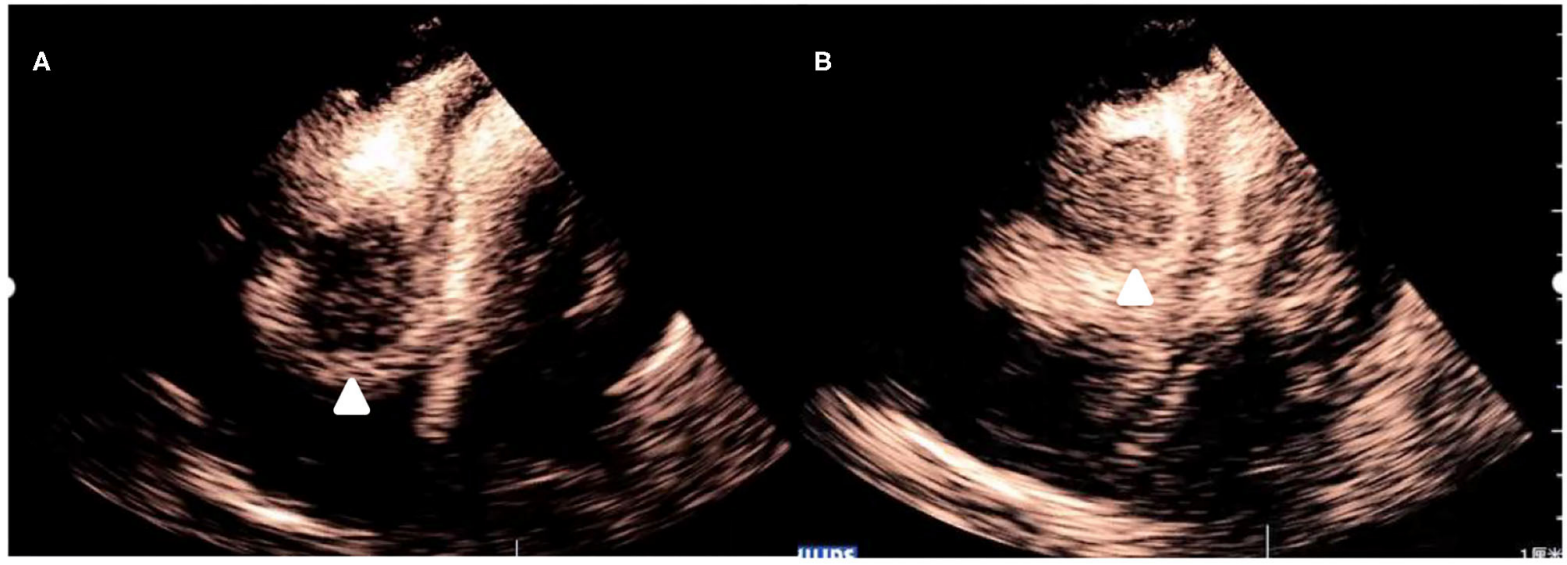
Myocardial contrast echocardiography images of the mass. **(A)** After administration of the contrast agent, there is partial enhancement of the mass. **(B)** After several cardiac cycles, there is an increase in enhancement of the mass.

**Figure 3:**
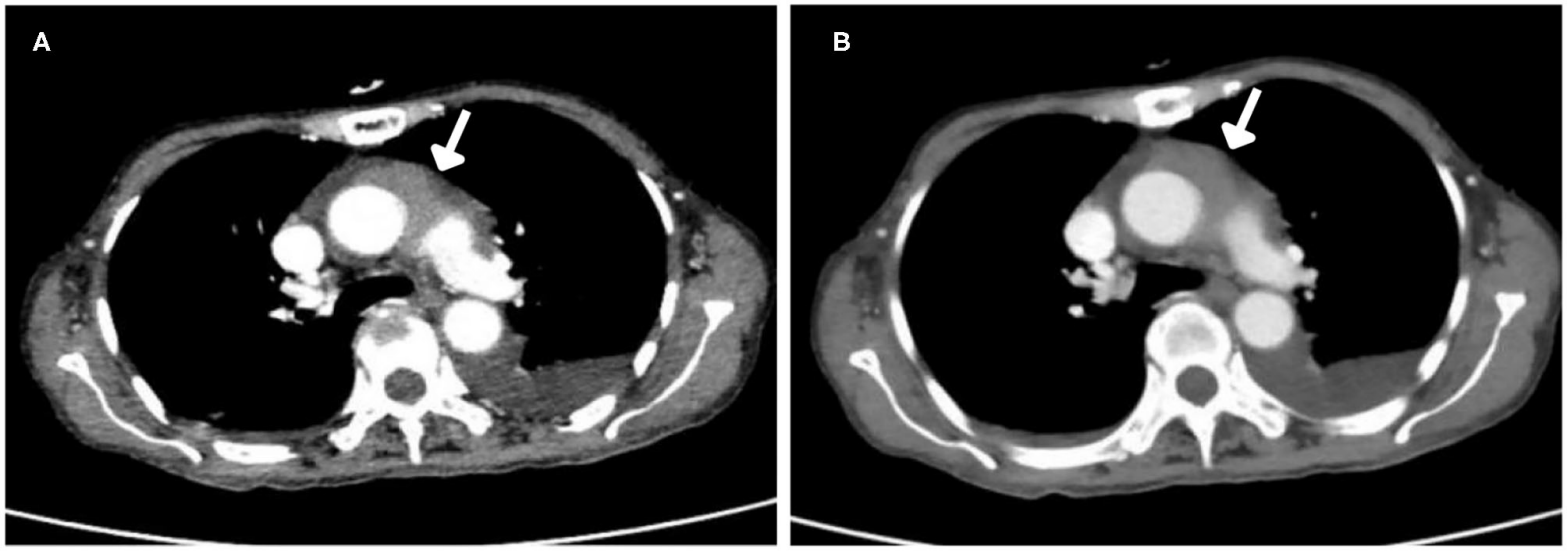
Computed tomography (CT) images. **(A)** Arterial phase. The mass was mildly enhanced in the arterial phase. **(B)** Venous phase. The mass enhancement was reduced in the venous phase.

**Figure 4:**
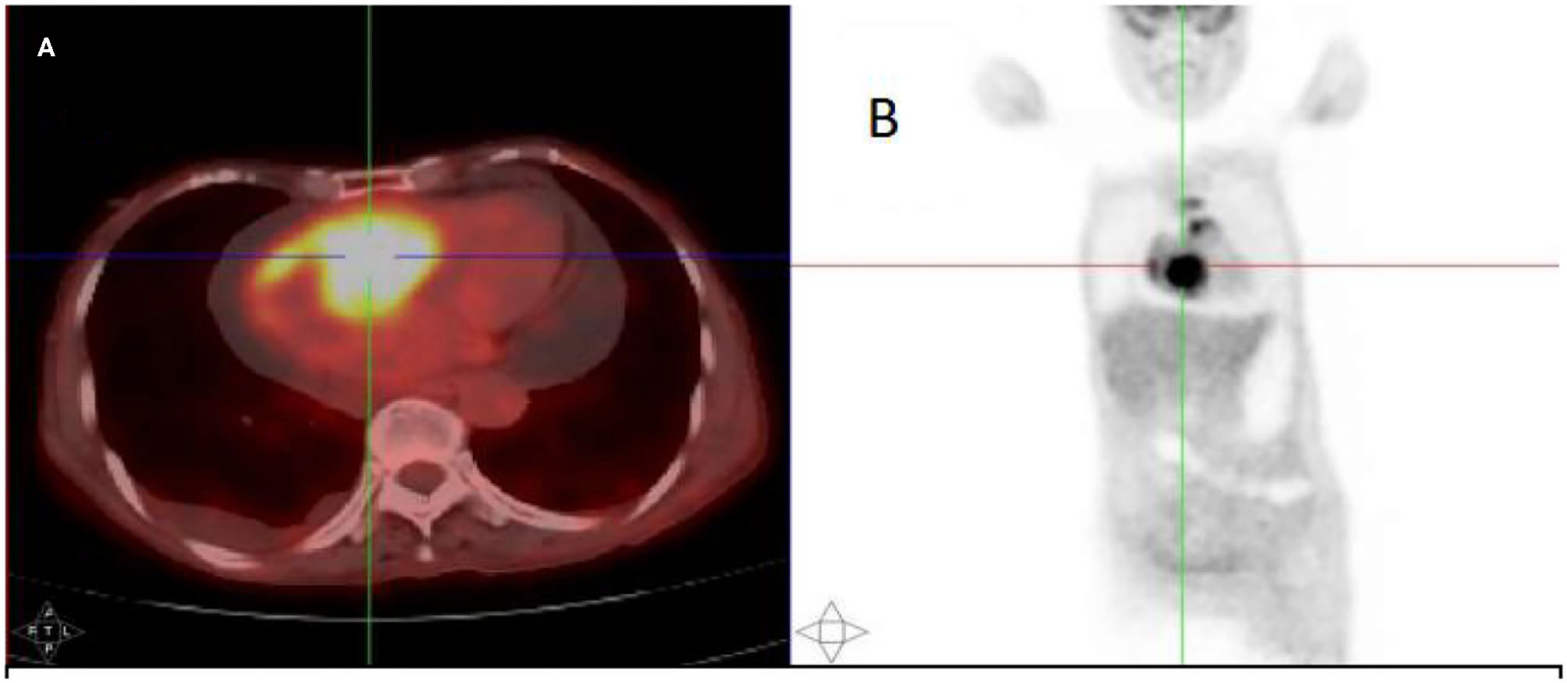
The 18F-fluorodeoxyglucose positron emission tomography (PET)/CT scan. **(A)** A mass in the right atrium with high radiotracer uptake. **(B)** Whole-body maximum-intensity projection PET images showed high radiotracer uptake in both the right atrium and aorta-ascending para-aorta.

After injection, the contrast agent rapidly filled the right atrium and the right ventricle, followed by the left atrium and the left ventricle. After several cardiac cycles, a large amount of scattered ultrasound contrast agent was observed in the right atrial mass (Figures 2A,B). Contrast-enhanced computed tomography (CT) revealed a mass near the aortic arch, which was mildly enhanced in the arterial phase (Figure 3A) and reduced in the venous phase (Figure 3B). When evaluating inflammation by positron emission tomography (PET)/CT (Figure 4), masses in the right atrium, the walls of the right atrium and the right ventricle were discovered to be non-homogeneously thickened. In addition, 18F-fluorodeoxyglucose uptake was increased. The ascending aorta–para-aortic soft tissue density mass and increased 18F-fluorodeoxyglucose metabolism indicated that both the right atrial and aorta-ascending–para-aorta tumors were malignant.

**Figure 5:**
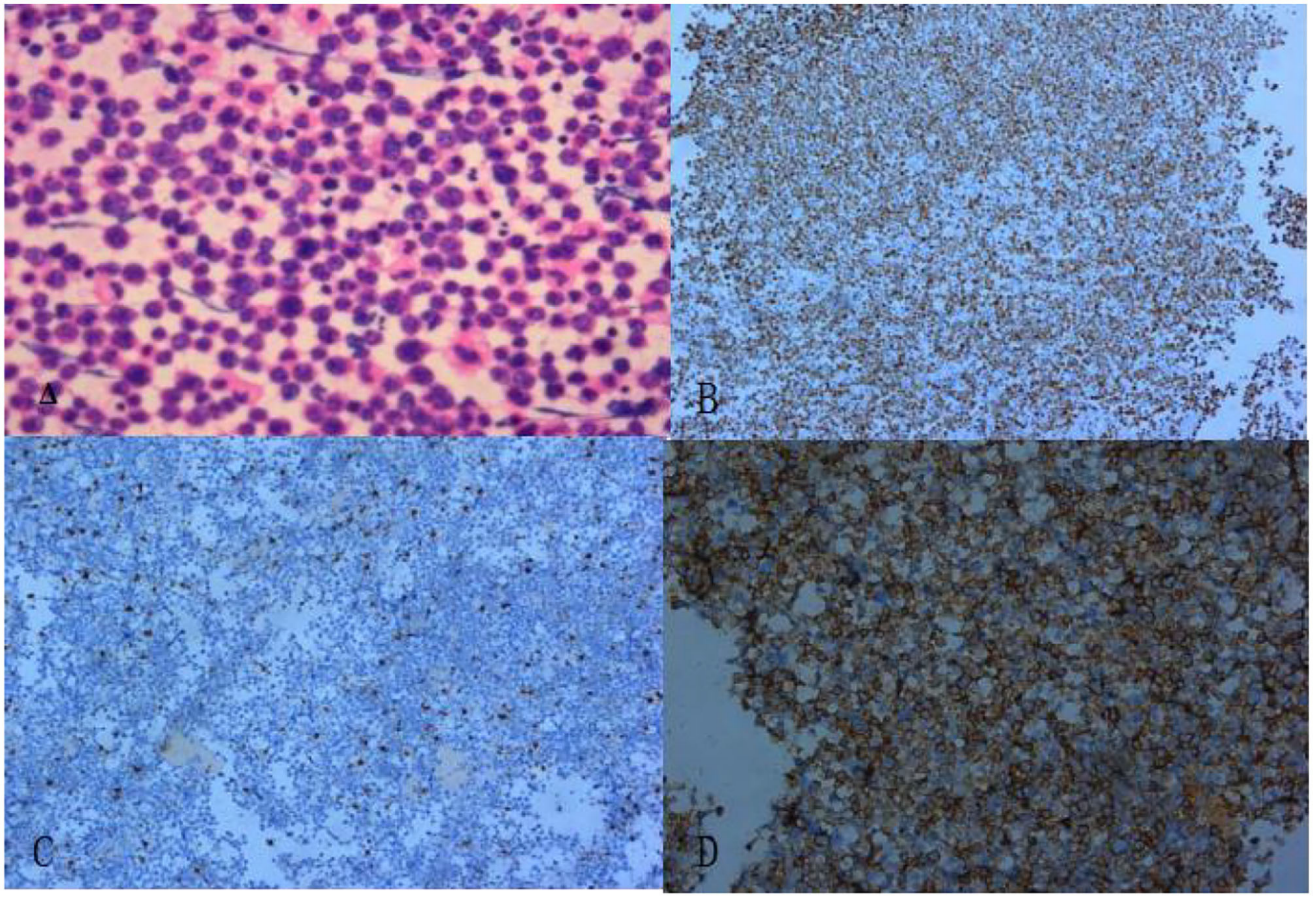
The pathological section revealed diffuse enlargement of lymphocytes. **(A)** Hematoxylin and eosin staining (× 200). **(B)** Ki-67 markers (+) (× 100). **(C)** CD3 markers (–) (× 100). **(D)** CD20 markers (+) (× 100).

Finally, a pathological examination of the cell mass in the pericardial effusion (Figure 5) diagnosed it as diffuse large B-cell non-Hodgkin's lymphoma. The immunohistochemical results were as follows: BCL-2 (+, 90%), CD19^+^, CD20^+^, CD5^+^, CD79a^+^, c-Myc (+, 30%), Ki-67 (+, 80%), and MUM-1^+^.

With the collection of multi-modality imaging data and histologic findings, the patient's heart mass was finally diagnosed as cardiac lymphoma which needed to be differentiated from other space-occupying cardiac lesions. Because it was a mass with a rich blood supply, while benign lesions or thrombi in the heart tend to have poor blood supply, it was considered to be a malignant mass on imaging and was confirmed as lymphoma by pathological examination.

The patient declined treatment and ultimately died 1 year later.

## Discussion

Primary cardiac lymphoma is described as an extranodal lymphoma involving only the heart and/or pericardium, presenting with cardiac symptoms that are accompanied by the development of the main tumor mass in the heart or pericardium (2). The latter definition is more applicable in clinical practice. Cardiac lymphoma is a malignant tumor which usually occurs in the right cardiac system, especially the right atrium which may, possibly, abnormally collect lymphatic fluid from the whole body (3). Its clinical manifestations are atypical and includes arrhythmia, chest pain, and fever (4). In the present case, a primary cardiac lymphoma located in the right atrium was discovered.

Although cardiac imaging can also provide useful information, the diagnosis of cardiac lymphoma depends on the pathology results. Chemotherapy is the preferred treatment of heart lymphoma (5). According to the World Health Organization classification, lymphoma with positive expressions of both C-Myc and Bcl-2 or Bcl-6 detected by immunohistochemistry is known as a double-expression lymphoma, which has a poor prognosis (6). In the present study, the patient had positive expressions of both c-Myc and Bcl-2. In such cases, early detection and treatment are very important for the health of the patient.

Coronary angiography is required to exclude coronary artery stenosis in patients being considered for cardiac surgery. Both CT and cardiac MRI are complementary imaging modalities to ECG. The CT scan is also important to show the extent of the lesion in the case of a potential differential diagnosis like renal cell carcinoma with spread *via* the inferior vena cava in the case of myxoma. Additionally, magnetic resonance imaging (MRI) has high spatial resolution and good tissue contrast. Hence, it can clarify the anatomical position relationship of each structure, divide the boundary between the tumor and normal tissue, and judge benign and malignant tumors by their behavioral characteristics, all of which are helpful in guiding the surgical treatment and prognosis evaluation. Although MRI is the gold standard for evaluating the heart's structure and function, ultrasonic cardiography is more convenient to perform and much more reasonably priced. In addition, conventional ECG can evaluate the effect of the mass on cardiac function and morphology. It is also able to identify space-occupying lesions, but it cannot effectively distinguish malignant lesions from benign ones. With the development of ultrasonic techniques, contrast-enhanced ultrasound (CEUS) is found to be capable of effectively and directly identifying the location, shape, and size of space-occupying lesions and discerning their relationship with surrounding tissues. Multi-modality imaging data are very helpful for the diagnosis of cardiac masses and for patient management.

Contrast-enhanced ultrasound is also important for diagnosing cardiac space-occupying lesions. To date, there have been few reports of cardiac lymphoma diagnosed by myocardial contrast ECG. Non-neoplastic heart lesions show no enhancement due to the lack of blood flow. Benign tumors show some sparse enhancement due to poor blood supply. Malignant tumors are rich in newly formed blood vessels. Therefore, they show significant enhancement in medical imaging procedures. Shimizu et al. (7) applied myocardial contrast ECG with 1.5-harmonic imaging to an atrioventricular groove tumor of a patient with malignant lymphoma. Contrast ECG initially showed a single central lesion and some patchy echogenic foci within the tumor, all of which were indicative of arterial components. Subsequently, the tumor was homogenously enhanced, reflecting parenchymal hyperperfusion. Although the initial tumor-enhancement pattern was not observed in the present case, uniform enhancement and high perfusion were present. The mass was misdiagnosed as benign during a traditional ultrasound examination. However, CEUS indicated a malignancy. Thus, the ultrasound enhancement result was more accurate. CEUS can assist in clarifying benign or malignant lesion manifestation and is recommended for application in clinical practice. However, perfusion patterns specific to each tumor have yet to be elucidated (8) and require further exploration in the future.

Detailed collection of non-invasive data and histologic findings suggest an advantage of multi-modality imaging and may ensure more effective management of patients with cardiac tumors.

## Data Availability Statement

The original contributions presented in the study are included in the article/supplementary material, further inquiries can be directed to the corresponding author/s.

## Ethics Statement

Written informed consent was obtained from the individual(s) for the publication of any potentially identifiable images or data included in this article.

## Author Contributions

XXJ contributed to the conception of the case report. DYY contributed to the manuscript writing. TNW, LLL, and LNG contributed to the clinical data collection. FJL contributed to the pathological diagnos. All authors contributed to the article and approved the submitted version.

## Funding

This study was funded by Hainan Provincial Science and Technology Special Fund (ZDYF2019136 and ZDYF2020140).

## Conflict of Interest

The authors declare that the research was conducted in the absence of any commercial or financial relationships that could be construed as a potential conflict of interest.

## Publisher's Note

All claims expressed in this article are solely those of the authors and do not necessarily represent those of their affiliated organizations, or those of the publisher, the editors and the reviewers. Any product that may be evaluated in this article, or claim that may be made by its manufacturer, is not guaranteed or endorsed by the publisher.
